# THE EFFECTS OF CHRONIC PAIN AND LONELINESS ON FUNCTIONING AMONG LATINO AND WHITE OLDER ADULTS

**DOI:** 10.1093/geroni/igz038.2169

**Published:** 2019-11-08

**Authors:** David Camacho, Maria P Aranda, Denise Burnette, Ellen Lukens

**Affiliations:** 1 Columbia University, New York, New York, United States; 2 University of Southern California, Los Angeles, California, United States; 3 Virginia Commonwealth University, Richmond, Virginia, United States

## Abstract

The detrimental effects of loneliness and chronic pain on functioning in later life are well documented, yet there is little evidence of whether these patterns hold across racially diverse older adults. Guided by the Biopsychosocial Model of Assessment, Prevention, and Treatment of Chronic Pain, we used data from Waves 2 and 3 of the National Social Life, Health, and Aging Project (NSHAP) to examine the additive and interactive effects of loneliness and chronic pain on Elemental and Instrumental Activities of Daily Living (ADLs & IADLs) among a sample of 1046 Latino and White adults aged 50 and over. Using linear regression analyses, our final models (Adjusted R-squares: .316 & .304) included demographic characteristics, physical and mental health, medication, health behaviors and social factors. In this sample, approximately 33% experienced chronic pain, 50% reported at least transitory loneliness and 22% experienced both. Neither loneliness nor chronic pain was independently associated with functioning impairment. However, these two factors in combination were associated with lower scores on ADLs and I-ADLs. In addition, Latinos who reported chronic pain were more likely to report lower scores on ADLs only. Results highlight variations in the detrimental effects of loneliness and chronic pain for white and Latino elders. Findings suggest the need for interventions that address chronic pain and loneliness simultaneously. Future studies should examine how culturally-grounded experiences of loneliness and chronic pain may contribute to worsening of functioning among diverse groups of Latino elders.

